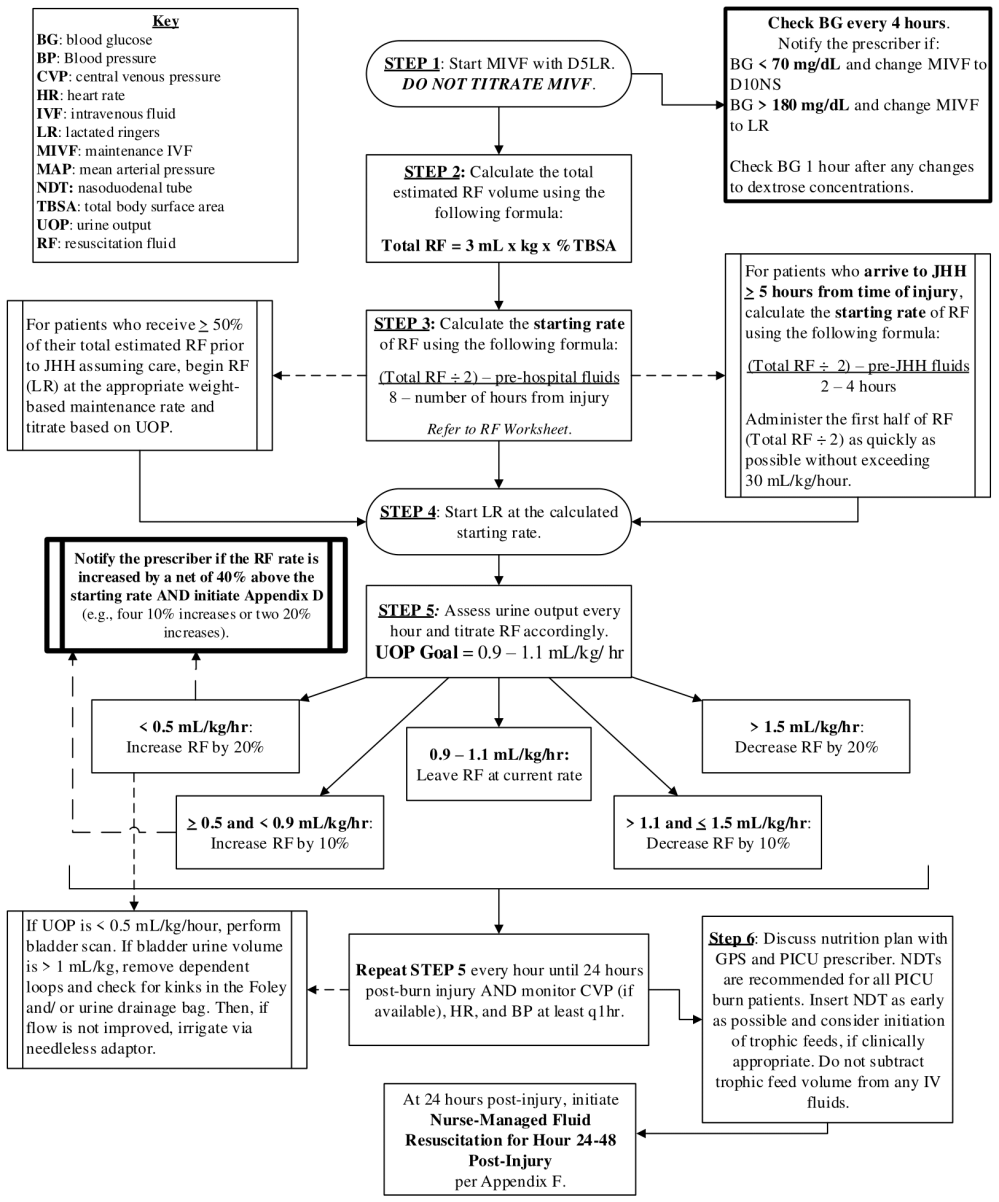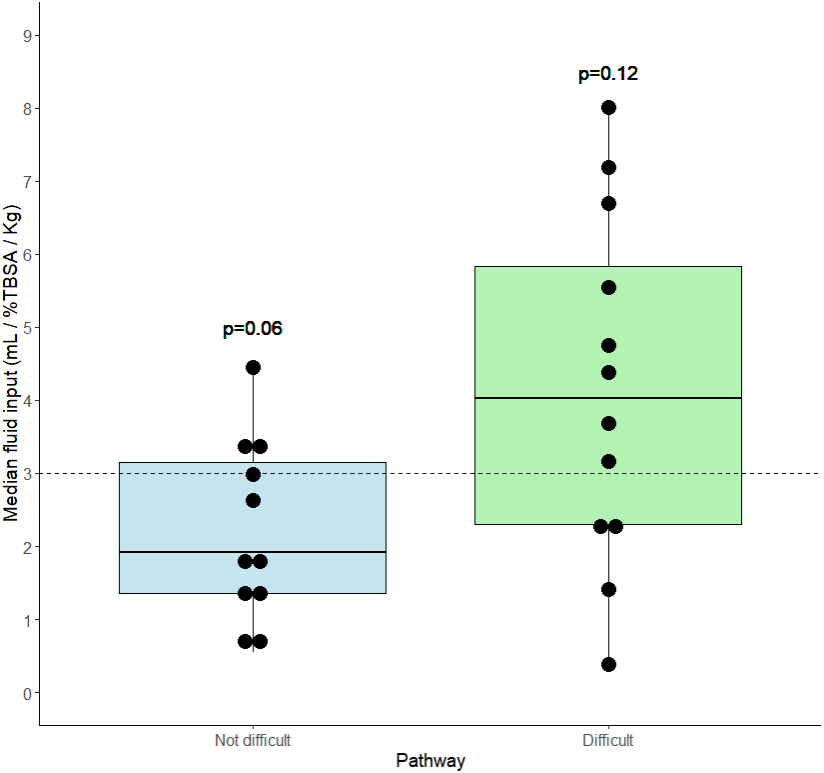# 88 Fluid Resuscitation in Pediatric Burn Patients: An Institutional Protocol Assessment

**DOI:** 10.1093/jbcr/iraf019.088

**Published:** 2025-04-01

**Authors:** Stephanie Morgenstern, Charbel Chidiac, Kristin Wharton, Christina Fellner, Alejandro Garcia

**Affiliations:** Johns Hopkins Hospital; Johns Hopkins Pediatric Burn Center; Johns Hopkins Children’s Hospital; Johns Hopkins Children’s Hospital; Johns Hopkins University

## Abstract

**Introduction:**

Burn injuries in children require careful management, particularly regarding fluid resuscitation to prevent shock while maintaining tissue perfusion. In 2016, our institution developed a nurse-driven fluid resuscitation pathway (Figure 1). Our study evaluates the effectiveness and safety of our institution’s fluid resuscitation protocol in pediatric patients with severe burns.

**Methods:**

We conducted a retrospective review of pediatric burn patients admitted at our institution from 2016-2022. Patients < 10Kg with a total body surface area (TBSA) ≥15% and those ≥10Kg with TBSA≥20% were included. We compared the total resuscitation rate administered during the first 24hours, calculated as (Normal Saline+Albumin+Lactated Ringer)/%TBSA/Kg, with the modified Parkland formula rate (3 mL/%TBSA/Kg/Day).

**Results:**

Among 23 patients, median age was 2 years. 12 (53%) patients were difficult to resuscitate. Median weight was 15.0 kg (IQR 11.5-32.4) with a median TBSA affected of 22%. 13 (56.5%) had full-thickness burns, 14 (60.9%) were scald injuries, and 3 (13.0%) were abuse. The mean resuscitation fluid rate administered was 2.99 mL/%TBSA/kg, not significantly different from the Parkland formula rate (p=0.61). Patients without difficult resuscitation received fluids at 1.91 mL/%TBSA/kg (p=0.06), whereas those with difficulties had a rate of 4.03 mL/%TBSA/kg (p=0.12) (Figure 2). Mean urine output was 1.74±0.89 mL/Kg/hour, and pulmonary edema developed in 5 (21.7%) patients within the first 7 days. Median ICU lengths of stay was 11 days, with no fatalities observed.

**Conclusions:**

Our study demonstrates that our institutional fluid resuscitation pathway for pediatric burn patients is effective and safe, aligning well with the modified Parkland formula and managing resuscitation challenges without increasing complications. This underscores the value of nurse-led interventions in optimizing care and improving outcomes for pediatric burn patients.

**Applicability of Research to Practice:**

The implementation of a nurse-driven protocol standardizes fluid resuscitation, ensuring adherence to guidelines while allowing flexibility for individual patient needs. This approach enhances bedside decision-making, reduces complications from fluid mismanagement, and empowers nursing staff in critical care. This protocol can be adopted by other institutions with the hope to improve pediatric critical burn care.

**Funding for the Study:**

N/A